# Rhabdomyolysis: A Case Report of an Extrapulmonary Presentation of *Mycoplasma pneumoniae*

**DOI:** 10.5811/cpcem.2020.9.46024

**Published:** 2021-03-12

**Authors:** Michael Gulenay, V. Andres Sasson, Kevin Taylor

**Affiliations:** St. Lucie Medical Center, Palm Beach Consortium for Graduate Medical Education, Department of Emergency Medicine, Port St. Lucie, Florida

**Keywords:** Rhabdomyolysis, Mycoplasma pneumoniae, extrapulmonary manifestations

## Abstract

**Introduction:**

We present an unusual case of rhabdomyolysis secondary to *Mycoplasma pneumoniae* in a healthy 27-year-old female. *M. pneumoniae* is associated with numerous extrapulmonary manifestations, including acute hepatitis, Stevens-Johnson syndrome, and rhabdomyolysis. Most documented cases affect the pediatric population, with only five cases in adults reported in the literature.

**Case Report:**

The patient presented with complaints of myalgia and intractable cough. In this case the initial presentation demonstrated hypoxia requiring supplemental oxygen, and a creatine kinase of 7,646 units per liter (U/L) (reference range 26–192 U/L) with a peak of 29,427. During her hospitalization, she also remained persistently hypoxic for several days but ultimately was successfully weaned off all supplemental oxygen. She was discharged home after a seven-day hospitalization.

**Conclusion:**

This patient’s presentation of an insidious, upper respiratory infection along with the subsequent development of rhabdomyolysis with reactive antibodies to *M. pneumoniae* demonstrates a link between these two clinically important conditions.

## INTRODUCTION

*Mycoplasma pneumoniae* is the most common cause of community-acquired pneumonia in healthy young patients under age 40.^1^,^2^ It can affect people of any age group but is typically associated with ages 5–20.^1^, ^2^ It is transmitted from person to person via respiratory droplets and has a subtle and lingering presentation.^1^, ^2^ The incubation period is approximately 2–3 weeks and symptoms are characteristic of most viral infections. It is differentiated by the intractable and progressively worsening cough. It is worth noting that many patients who become infected with *M. pneumoniae* are asymptomatic.^1^,^2^ In rare circumstances patients develop extrapulmonary disease including dermatological, gastrointestinal, neurological, and musculoskeletal manifestations.^1^–^3^ Rhabdomyolysis is one of these rare manifestations; a literature review reveals that most cases affect the pediatric population and a handful of cases reported in healthy adults.^3^–^12^ We present an unusual case of rhabdomyolysis secondary to *M. pneumoniae* in a healthy young adult female with hypoxia and myalgia.

## CASE REPORT

A 27-year-old female with a pertinent medical history of tobacco use and intrauterine device implantation presented to the emergency department (ED) with a chief complaint of progressively worsening shortness of breath for two days. Associated symptoms included a non-productive cough, tactile fevers, diaphoresis, muscle aches, and chest pain when coughing. Symptoms were present for two weeks and were felt to be improving until four days prior. The patient was also evaluated in the ED the previous day and was prescribed prednisone and doxycycline for a diagnosis of atypical pneumonia. Additional review of symptoms was negative for hemoptysis, extremity pain or swelling, history of venous thromboembolic disease, or any travel.

Physical examination was significant for an anxious and diaphoretic female in moderate respiratory distress. She appeared uncomfortable and was in a tripod position. Auscultation revealed bilaterally diminished breath sounds with mild expiratory wheezing. The rest of the examination was unremarkable except for tachycardia. Initial vital signs were a temperature of 98.1° degrees Fahrenheit (36.7° Celsius), pulse of 107 beats per minute, blood pressure of 134/75 millimeters of mercury, respiratory rate of 24 breaths per minute, and pulse oximetry of 91% on two liters oxygen via nasal cannula.

In view of the patient’s respiratory distress, she was given nebulized ipratropium bromide and albuterol, in addition to an intravenous (IV) anxiolytic. This resulted in an improvement in her respiratory effort and work of breathing. The hypoxia improved through the implementation of 100% oxygen via a non-rebreather mask, which was subsequently converted to a high-flow nasal cannula. Oxygen saturations stabilized and remained greater than 95% after she received the above treatments. The patient was empirically treated with IV ceftriaxone and doxycycline and two liters of IV normal saline. Both plain chest radiograph and computed tomography angiography of the chest were negative for acute findings.

Laboratory findings were significant for leukocytosis of 15.4 x 10^3^ microliters (μL) (reference range 3.6–11 × 10^3/^/μL); lactic acid of 2.3 millimoles per liter (mmol/L) (reference range 0.4 – 2.0 mmol/L); C-reactive protein of 5.12 milligrams per deciliter (mg/dL) (ref range 0.00–0.30 mg/dL); creatine kinase of 7,646 units/L (ref range 26–192 U/L); and creatine kinase MB (CK-MB) of 42.0 nanograms per milliliter (ng/mL) (ref range 0.0–5.6 ng/mL). Rapid influenza diagnostic testing was negative. *M. pneumoniae* antibody testing and viral panel studies were also collected but did not result until later in the admission. Patient denied illicit drug use, including methamphetamine abuse, which was confirmed by a negative urine drug screen. She was admitted to the intensive care unit and treated with aggressive IV hydration therapy, supplemental oxygen, and antibiotics.

**Figure 1:**
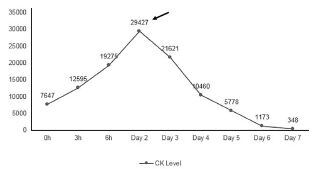
Creatine kinase (CK) trend during hospitalization. The peak CK value is denoted by the arrow. All values measured in units per liter (U/L) with a reference range of 26–192 U/L.

**Figure 2:**
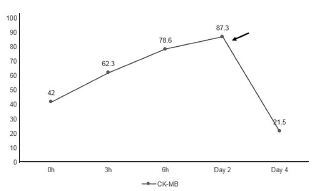
Creatine kinase MB (CK-MB) trend during hospitalization. The peak CK-MB value is denoted by the arrow. All values measured in nanograms per milliliter (ng/mL) with a reference range of 0.0–5.6 ng/mL.

During her hospitalization, both the serum creatine kinase and CK-MB levels were monitored serially and continued to rise with peaks on day two of 29,427 U/L and 87.3 ng/mL, respectively. These levels gradually trended down to a normal reference range by day seven and day four, respectively (Figures 1 and 2). Renal function was unaffected and remained normal with an initial creatinine of 0.76 mg/dL and a peak of 0.79 (reference range 0.43 to 1.13 mg/dL). Relevant admission findings included reactive immunoglobulin M antibodies to *M. pneumoniae*. She also remained persistently hypoxic for several days but ultimately was successfully weaned off all supplemental oxygen and discharged home after a seven-day hospitalization.

## DISCUSSION

Rhabdomyolysis is a rare extrapulmonary manifestation of *M. pneumoniae*, and literature review reveals it to be infrequently described in healthy adults.^3^–^7^ The rod-shaped bacterium is excreted from the respiratory tract weeks after the acute infection and has been associated with acute hepatitis, immune thrombocytopenic purpura, Stevens-Johnson syndrome, and transverse myelitis.^1^,^2^ The pathogenicity of *M. pneumoniae* has been linked to the activation of inflammatory mediators such as cytokines.^1^,^2^ However, the mechanism of muscle damage due to infection has not been established and remains unclear. One theory postulates direct invasion and toxic degeneration of the muscle fibers.^1^,^2^

**CPC-EM Capsule**

What do we already know about this clinical entity?

Mycoplasma pneumonia*e (*M. pneumoniae*) has been linked with numerous extrapulmonary conditions and it has been postulated that the activation of inflammatory mediators such as cytokines leads to the muscle damage.*

What makes this presentation of disease reportable?

*Rhabdomyolysis secondary to* M. pneumoniae* in a healthy young adult is a rare presentation. A literature review reveals that most cases affect the pediatric population, rather than healthy adults.*

What is the major learning point?

*As emergency physicians, we must maintain a wide differential and high suspicion to avoid missing potentially important secondary diagnoses critical to favorable patient outcome.*

How might this improve emergency medicine practice?

*The case highlights the need to maintain suspicion for life-threatening causing extrapulmonary manifestations in patients with straightforward-appearing upper respiratory infections.*

The pathophysiology of rhabdomyolysis, however, is well understood and involves the release of intracellular components secondary to skeletal muscle necrosis.^1^,^2^ This results in large quantities of potentially toxic intracellular substances being released into the plasma including creatinine kinase and myoglobin. The myoglobin precipitates into the glomerular filtrate resulting in nephrotoxicity and acute kidney injury. It clinically presents as myalgia and dark urine and is diagnosed when the creatine kinase level is five times the upper limit of normal.^1^,^2^ There are numerous causes of rhabdomyolysis including trauma, extreme exertion, heatstroke, medication side effect, illicit drug use, and infections.

Infectious causes include both viral and bacterial pathogens such as influenza A/B, coxsackievirus, *M. pneumoniae*,* Legionella* species, and *Salmonella* species.^1^–^2^,^11^–^12^ This patient’s presentation of an insidious upper respiratory infection along with the subsequent development of rhabdomyolysis with reactive antibodies to *M. pneumoniae* demonstrates a link between these two clinically important conditions. It is noteworthy to mention that the recent upper respiratory symptoms and the new complaints of chest pain and shortness of breath raised initial concern for myopericarditis. This concern was mitigated by normal serial troponins and transthoracic echocardiogram.

## CONCLUSION

The case highlights the need to maintain suspicion for life-threatening risk and morbidity causing extrapulmonary manifestations in patients with otherwise straightforward-appearing upper respiratory infections. The diagnosis can be challenging, such as in this case, as patients may not initially complain of muscle soreness or cramping, which is often representative of rhabdomyolysis. The extrapulmonary presentation of rhabdomyolysis secondary to *Mycoplasma pneumoniae* is rare; to our knowledge there are only five previously documented cases in healthy adults.^3^–^7^ As emergency physicians, we must maintain a wide differential and high suspicion to avoid missing potentially important secondary diagnoses critical to favorable patient outcome.
